# c-*myc* and N-*myc* promote active stem cell metabolism and cycling as architects of the developing brain

**DOI:** 10.18632/oncotarget.116

**Published:** 2010-06-04

**Authors:** Alice Wey, Paul S. Knoepfler

**Affiliations:** ^1^Institute of Pediatric Regenerative Medicine, Department of Cell Biology and Human Anatomy, University of California Davis School of Medicine, Shriners Hospital For Children Northern California, Sacramento, CA 95817

**Keywords:** c-myc, N-myc, brain tumors, stem cells, metabolism, mitosis, pluripotency

## Abstract

*myc* genes are associated with a wide variety of human cancers including most types of nervous system tumors. While the mechanisms by which *myc* overexpression causes tumorigenesis are multifaceted and have yet to be clearly elucidated, they are at least in part related to endogenous *myc* function in normal cells. Knockout (KO) of either c-*myc* or N-*myc* genes in neural stem and precursor cells (NSC) driven by nestin-cre impairs mouse brain growth and mutation of N-*myc* also causes microcephaly in humans in Feingold Syndrome. To further define *myc* function in NSC and nervous system development, we created a double KO (DKO) for c- and N-*myc* using nestin-cre. The DKO mice display profoundly impaired overall brain growth associated with decreased cell cycling and migration of NSC, which are strikingly decreased in number. The DKO brain also exhibits specific changes in gene expression including downregulation of genes involved in protein and nucleotide metabolism, mitosis, and chromatin structure as well as upregulation of genes associated with differentiation. Together these data support a model of nervous system tumorigenesis in which excess *myc* aberrantly locks in a developmentally active chromatin state characterized by overactive cell cycling, and metabolism as well as blocked differentiation.

## INTRODUCTION

*myc* is well-known for its role in tumorigenesis when overexpressed and N-*myc* (MYCN) is most strongly associated with primitive “blast” nervous system tumors neuroblastoma, medulloblastoma, retinoblastoma, and glioblastoma [1-11]. However, at physiological levels *myc* genes are important regulators of many aspects of normal cell behavior including metabolism and cycling (reviewed in [12]). *myc* genes encode members of the basic-helix-loop-helix zipper (bHLHZ) transcription factor superfamily, but Myc proteins are very atypical bHLHZ factors in the diversity of their functions. They can activate and repress the transcription of specific protein coding genes, influence expression of miRNA and rRNA, drive DNA synthesis, and globally influence chromatin structure. More recently *myc* has been shown to relieve transcriptional pausing in embryonic stem cells (ESC) through a mechanisms involving PTEF-b [13], but in many cases the mechanisms by which *myc* achieves its diversity of functions are not well understood. One theory is that Myc's wide range of functions is linked to its widespread euchromatic function associated with specific histone modifications such as acetylation of lysine 9 and methylation of lysine 4 of histone H3 [14-19].

There is growing evidence of key roles for *myc* genes at endogenous levels in both somatic stem cells such as NSC and ESC. Constitutive knockout (KO) of c-*myc* or N-*myc* causes embryonic lethality around midgestation [20, 21]. Conditional disruption of N-*myc* in NSC severely disrupts murine brain growth, particularly that of the cerebellum, while a similar KO of c-*myc* moderately impairs growth [22, 23]. Disruption of either c-*myc* or N-*myc* or both in hematopoietic stem cells (HSC) also alters their normal biological functions, affecting survival and self-renewal [24, 25]. *myc* genes also are involved in the production of induced pluripotent stem (iPS) cells (reviewed in [26]) [27-31]. While exogenous *myc* is not formally required for the process [32, 33], it dramatically enhances the efficiency and in its absence its function is likely supplanted by endogenous *myc.* During iPS cell formation, Myc represses differentiation-associated genes [34] and may not have a key role in directly maintaining expression of pluripotency factors. However, in neuroblastoma some pluripotency genes such as lif, lin28b, Klf2, and Klf4 are N-Myc targets for activation, while a subset of these genes is also regulated in NSC by N-*myc* [35]. Another role that Myc may play in pluripotency is maintenance of the high levels of cellular metabolism, including protein (reviewed in [37]) and DNA [38] synthesis, observed in highly pluripotent cells.

Perhaps because of the importance of *myc* genes in normal cellular biology and their ability to cause cancer when in excess, cells have evolved systems to maintain normal total cumulative *myc* RNA and Myc protein levels. These include cross-regulation, redundancy and compensation between the 3 main *myc* genes – c-, N-, and L-*myc* – as well as *myc*-trigged apoptosis when in excess. Myc protein stability is also tightly controlled (reviewed in [39]). Conditional double knockout (DKO) of c- and N-*myc* in hematopoietic stem cells yields a far more severe phenotype than disruption of either gene alone, suggesting additive or redundant roles [25]. A large degree of redundancy is also supported by the knockin of N-*myc* into the c-*myc* locus largely rescuing the loss of c-*myc* [40]. The prevailing theory is that what is most critical is the total level of all *myc* gene expression in each cell. Despite fairly ubiquitous expression in the developing brain and some other regions of the embryo, L-*myc* constitutive KO was reported to have no phenotype at all [41]. One notion is that this lack of apparent phenotype was due to the continued presence of N-*myc* and perhaps c-*myc*, which could fulfill the roles of L-*myc* in its absence.

During neurogenesis, N-*myc* plays a particularly important role in NSC to direct brain growth and development [22, 42], consistent with its fairly widespread expression pattern. N-*myc* is also essential for normal eye and neural retina development [43, 44]. L-*myc* is also fairly widely expressed, particularly in the early midbrain, which suggests it may function in the brain despite no reported KO phenotype. Although c-*myc* expression has been less clearly defined, it appears to be more restricted than that of L- and N-*myc*. Overexpression and c-*myc* KO studies in rat neurospheres support a crucial role for *myc* genes overall in NSC function and suggested functional ties to p53/Arf [45], but the relevance for *in vivo* neurogenesis and tumorigenesis remains unclear. The molecular basis of the c- and N-*myc* nestin-cre driven brain phenotypes is also not completely clear, but in the N-*myc* knockout changes in cyclin D2 and cyclin dependent kinase inhibitors such as p18 and p27 were evident suggesting cell cycle regulation plays a role [22, 46], including in the cerebellum. Impeding our understanding is the fact that no unbiased global analysis of gene expression has been conducted for any Myc transcription factors using a loss of function model in the nervous system. Upstream of N-*myc* are several potential signaling pathways including Shh in the cerebellum and in cerebellar granule neural progenitors (CGNP) [23, 47], while N-*myc* also appears to be a critical part of a recently defined pathway DLL3 and Notch signaling in which N- Myc protein is targeted by the Huwe1 ubiquitin ligase [11, 48].

To address the endogenous functions of c- and N-*myc* in brain growth, we created a nervous system specific double c- and N-*myc* KO (DKO) mouse by crossing doubly homozygously *myc* floxed mice with nestin-cre, which is expressed specifically in NSC. The *myc* DKO mice have a nervous system phenotype much more severe than either single *myc* KO alone, which is ultimately lethal. In terms of mechanisms underlying the phenotype, we found evidence that *myc* transactivates key gene expression programs in NSC including that of metabolic genes essential for normal stem and precursor fate during neurogenesis, chromatin regulatory genes, and mitosis-related genes. In addition *myc* appears to repress expression of genes associated with differentiation. Together these findings support the model that excess *myc* causes nervous system tumors by “locking in” a chromatin-based developmental program in NSC characterized by rapid cell cycling, high cellular metabolism, and blocked differentiation.

## RESULTS

### Double knockout (DKO) of c- and N-*myc* in NSC driven by nestin-cre causes a striking impairment of embryonic brain growth

**Figure 1.**
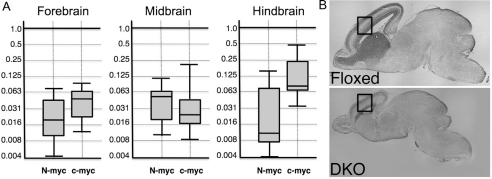
Nestin-Cre mediates potent reductions in both c- and N-myc expression levels throughout the brain causing dramatically impaired growth of the fore- and hindbrain, but not the midbrain (A) q RT-PCR for c- and N-*myc* expression. RNAs isolated from microdissected E17.5 control (N=4) and DKO (N=3) fore-, mid-, and hindbrains were used for q RT-PCR. Error bars are standard deviations. Mean levels in controls were set as 1.0. Median values are indicated by horizontal bars within vertical rectangles representing the range. p values were < 0.005 in each case. (B) Nissl stained E17.5 sagittal sections of doubly homozygously floxed littermate control and DKO (doubly homozygously floxed nestin-cre+). Boxed regions are shown at higher magnification in Fig. 2A.

Control (c-*myc* flox/flox; N-*myc* flox/flox) and DKO (c-*myc* flox/flox; n-*myc* flox/flox nestin-cre+) embryos were produced by timed matings. Embryonic brains were isolated at different stages and analyzed. At E14.5, embryonic brains were microdissected into fore-, mid-, and hindbrains followed by RNA isolation. q RT-PCR was conducted for c- and N-*myc*. All three main regions of the embryonic brain exhibited pronounced decreases in expression of both *myc* genes. c- and N-*myc* levels were reduced by approximately 20 and 50-fold in the forebrain, 40 and 20-fold in the midbrain, and 15 and almost 100-fold in the hindbrain, respectively (Fig. 1A). In terms of phenotype, the E17.5 brain overall was dramatically reduced in size, particularly in the forebrain/neocortex (Fig. 1B). The DKO hindbrain also exhibited a growth phenotype, albeit somewhat less severe at this stage. Surprisingly, the growth of the DKO midbrain was not significantly affected despite the strong reductions in c- and N-*myc* therein, suggesting midbrain growth prior to midgestation is largely independent of c- and N-*myc*. In support of this notion, the DKO adult midbrain was also largely normal (not shown).

### *myc* family expression patterns in the embryonic brain correlate with the general domain specificity of *myc* knockout phenotypes

We have previously observed high levels of N-Myc protein expression in the developing forebrain and hindbrain/cerebella [22]. These findings correlated with E15.5 data from the brain gene expression website: http://www.stjudebgem.org/web/mainPage/mainPage.php (Supplemental Fig. S1). While c-*myc* expression at the protein or RNA levels is difficult to detect, there are hints of low-level expression in a number of regions including the developing cortex. N-*myc* is fairly ubiquitously expressed, but with relatively lower or absent expression in some midbrain regions and very high levels in the neocortex and the developing cerebellum. L-*myc*, despite no reported KO phenotype, has particularly high expression in the ventricular zone (VZ) of the embryonic midbrain supporting the notion that L-*myc* may be the central driver of midbrain development, potentially explaining the absence of any clear midbrain phenotype in the c- and N-*myc* DKO.

### Expression of c- and N-*myc* in NSC is essential for normal architecture of the late embryonic cortical wall

**Figure 2.**
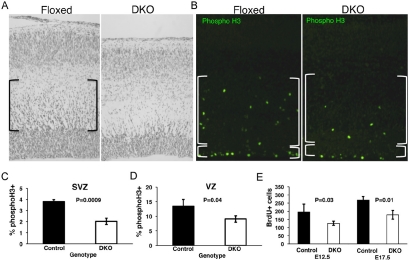
Loss of c- and N-*myc* disrupts cortical wall development with a particularly pronounced affect on the SVZ (A) Nissl stained E17.5 sagittal sections of cortical wall. Brackets indicate the normal SVZ, largely absent in the DKO. (B) Immunostaining for the mitotic marker phospho-H3 (green) in control and DKO E17.5 sections. Large and small brackets indicate SVZ and VZ respectively. (C) and (D) Quantification of percent mitotic/phosphoH3+ cells in the SVZ and VZ. (E) Quantification of S phase/BrdU+ cells in the entire cortical wall in control and DKO. Error bars are standard deviations. P values are indicated.

Because the DKO E17.5 cortex exhibited a dramatic overall phenotype visible to the eye, we examined it further by microscopic histological analysis of Nissl stained sagittal sections (Fig. 2A). The DKO cortical wall has a number of phenotypic characteristics including reduced overall thickness. Also evident was a hypocellular VZ and an apparent near complete absence of subventricular zone (SVZ; brackets in the floxed control) cells. These data suggest very few precursors are migrating through and out of the SVZ in the DKO, however the cortical plate at this stage is only modestly reduced in number and not at all in thickness. Together these findings suggest precocious formation of the cortical plate earlier than normal during differentiation.

### Loss of c- and N-*myc* strongly reduces cell cycling, especially mitosis in the SVZ

To examine the potential effects of the *myc* DKO on cell cycling we stained for BrdU for S phase cells and phosphoH3 for M phase cells. The DKO cortical wall exhibited a pronounced decrease in the percentage of M phase cells (Fig. 2B), approximately 2-fold in the SVZ and 30% in the VZ (Figs. 2C-D). The more pronounced loss of mitotic cells in the SVZ is consistent with the notion of decreased migrating progenitors suggested by the Nissl staining. The absolute number of mitotic cells was even more dramatically decreased given the hypocellularity of the DKO cortical wall. We also observed a statistically significant loss of BrdU+ cells at all stages examined including as early as E12.5 (Supplemental Fig. S2A, Fig. 2E) and at E17.5.

### Loss of c- and N-myc alters cell fate in the SVZ and VZ, consistent with a substantial decrease in stem and progenitors

**Figure 3.**
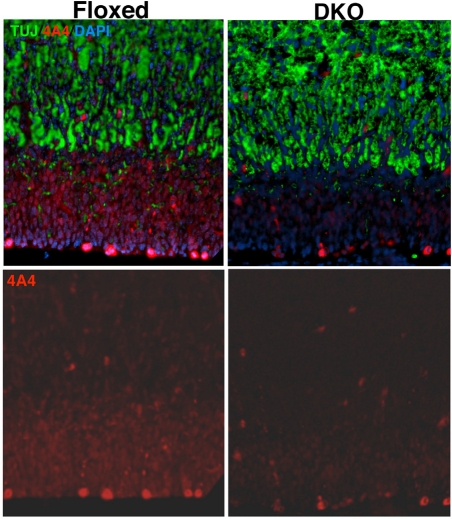
c- and N-*myc* are required to maintain normal NSC cell cycling in the forebrain E17.5 Sagittal sections of control and myc DKO cortical walls were stained with TUJ (green), 4A4 (red) and DAPI (blue). 40x magnification.

To examine the identity of cells in the cortical wall, we stained for 4A4 (a marker of stem and progenitors) and neural specific B-tubulin III (TUJ) in control and DKO sections (Fig. 3). The absolute size of the TUJ+ subdomain was not substantially altered in the DKO at E14.5 or at E17.5. However, because the overall cortical wall was greatly reduced in thickness in the DKO, the relative proportion of the wall that was TUJ+ was strikingly increased in the DKO (E17.5, not shown; E14.5, Supplemental Fig. S4). Supporting the notion of reduced progenitors, particularly those that are migrating, we saw decreased 4A4+ cells in the DKO (Fig. 3). The decrease in 4A4+ stem and precursor cells was not due to impaired cell survival as TUNEL staining indicated no clear different in apoptosis between control and DKO at E17.5 or at E12.5 (Supplemental Fig. S5; not shown). Interestingly, we found that while anti-4A4 (phospho vimentin) most brightly stains basal stem/precursors immediately adjacent within the VZ, cells in the TUJ- regions of the SVZ also stained 4A4+ (albeit with only moderately intense staining). Such staining is nearly completely absent from the DKO (Fig. 3 bottom). Thus, the SVZ DKO cells are both TUJ and 4A4 negative as well as mostly negative for S and M phase of the cell cycle, suggesting that they are in an abnormal developmental state and location due to the loss of *myc*.

### c- and N-*myc* maintain expression of genes involved in most aspects of cellular metabolism, chromatin regulatory factors, and mitosis

**Figure 4.**
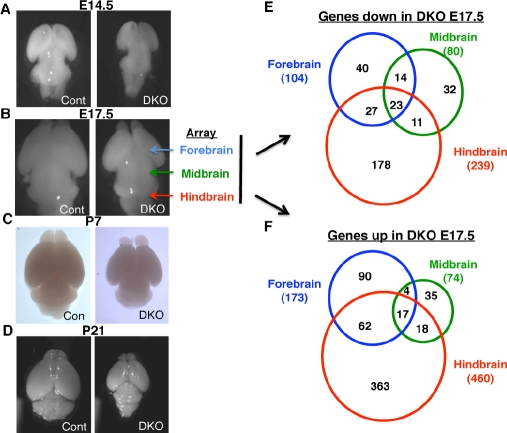
*myc* DKO whole brain and gene expression phenotypes Brains of indicated genotypes (DKO and littermate doubly floxed cre negative controls) were microdissected and photographed from above (A-D). For expression microarrays, RNA was isolated from the indicated domain (fore, mid, or hindbrain; blue, green, and red respectively) in biological triplicate (DKO) or quadruplicate (control, doubly floxed Cre-). (E-F) Venn diagrams indicate the number of genes whose expression was changed 1.3 fold or more in the indicate domain and the overlap.

To explore the mechanisms by which Myc proteins acting as transcription factors could regulate brain development and specific NSC functions such as cell cycling and fate, gene expression array studies were conducted on RNAs isolated from E17.5 control and DKO subdomains of the brain including forebrain, midbrain, and hindbrain/cerebellum (Fig. 4). Whole brains were first isolated from E14.5-P21 to compare overall severity of phenotypes (Fig. 4A-D). A phenotype was apparent as early as E12.5 and became progressively more severe. Interestingly, while tangential growth of the brain was only modestly reduced, rostro-caudal growth was severely impaired at all stages. Forebrain, midbrain, and hindbrain were microdissected from control (4 biological replicates) and DKO (3 biological replicates), RNA was produced, and used to probe promoter expression arrays (Fig. 4E-F). There was substantial overlap in the specific genes whose expression was altered in the DKO in the 3 domains, suggesting some conserved transcription functions for Myc in different subdomains of the brain. The magnitude of gene expression changes (total # of genes with increased or decreased expression) not surprisingly was directly linkable to the severity of the phenotype in a given domain. For example, midbrain, which exhibits little if any phenotype had far fewer expression changes. Interestingly no two domains were more similar to each other than the third, suggesting that although the midbrain had no obvious outward phenotype and relatively fewer gene expression changes, a loss of *myc* gene expression midbrain phenotype was occurring to some extent.

In addition to examining specific genes, complete lists of up or downregulated gene were subjected to ontological analysis to look for clusters of functionally related genes that are changed by loss of *myc* using the DAVID algorithm (http://david.abcc.ncifcrf.gov/). Focusing first on the candidate positively regulated Myc targets (genes downregulated in the DKO), the top scoring clusters were functionally related to ribosome biogenesis, protein translation, cellular metabolism, chromosomal binding proteins, and interestingly mitosis (Table 1). Of specific genes whose expression was altered by the DKO, most were verifiable by conventional RT-PCR and qRT-PCR (Supplemental Fig. S5A-B) and several downregulated genes, including Rpl23 and Bcat1 [50], are known *myc* target genes.

**Table 1.** Ontology of genes downregulated in DKO

Cluster Category	P-Value
**Forebrain**	
Ribosome	5.0 × 10^−13^
Nucleoside biosynthesis	8.1 × 10^−4^
	
**Midbrain**	
Actin	4.8 × 10^−4^
	
**Hindbrain**	
Ribosome	1.2 × 10^−32^
Chromosomal part	1.2 × 10^−8^
Nucleolus	4.8 × 10^−8^
DNA metabolism	2.9x 10^−7^
Mitosis	1.6 × 10^−6^

### Loss of *myc* leads to gene expression signatures of differentiation including ontological clusters of genes involved in patterning, morphogenesis, migration, and neurogenesis

There were many more genes upregulated by loss of *myc* than downregulated and correspondingly more functional ontological clusters (Table 2). Of the upregulated genes in the DKO, ontological analysis suggested that Myc most prominently repressed expression of genes associated with differentiation during neurogenesis and that in the DKO differentiation was occurring inappropriately at E17.5 in cells that would otherwise have remained NSC. Interestingly, we also found pronounced upregulation of a number of Wnt family members in the *myc* DKO hindbrain and N-*myc* has recently been implicated in cortical basal progenitors as a key effector of Wnt signaling [51]. The upregulation of Wnt signaling with loss of *myc* could reflect Myc repression of Wnt signaling in a negative feedback loop or alternatively be an indirect consequence of differentiation.

**Table 2.** Ontology of genes upregulated in DKO

Cluster Category	P-Value
**Forebrain**	
Signal	4.9 × 10^−11^
Branching morphogenesis	1.4 × 10^−6^
Cell Migration	3.2 × 10^−6^
Pattern Specification	1.9 × 10^−5^
Tube Development	4.4 × 10^−5^
Extracellular structure	4.7 × 10^−5^
Embryonic Organ Development	8.9 × 10^−5^
	
**Midbrain**	
Homeobox	3.6 × 10^−4^
	
**Hindbrain**	
Glycoprotein	9.3 × 10^−38^
Extracellular Matrix	4.2 × 10^−34^
Vascular Development	4.2 × 10^−16^
Tube Development	1.1 × 10^−12^
Cell Adhesion	1.1 × 10^−11^
Collagen	2.8 × 10^−9^
Heart morphogenesis	8.3 × 10^−8^
EGF-like region	8.5 × 10^−8^
Polysaccharide binding	1.0 × 10^−6^
Neurotransmitter transport	1.5 × 10^−6^
Fibronectin type III	2.7 × 10^−5^
Wnt signaling	1.2 × 10^−5^
Pattern Specification	3.3 × 10^−5^
Neuron differentiation	3.5 × 10^−5^
Hedgehog signaling	7.5 × 10^−5^
Bone Development	8.6 × 10^−5^
Cell motion	1.4 × 10^−4^
Ion Transport	3.3 × 10^−4^

### A subset of genes are consistently altered throughout the brain by loss of *myc*

When comparing the genes whose expression was changed in the 3 domains of the brain by loss of *myc*, it is important to note that a significant number had increased or decreased expression in all 3 domains (Supplemental Fig. S6). These genes therefore have the strongest link with *myc* in overall brain development. Amongst the shared downregulated genes (putative positively Myc regulated genes), although many are non-annotated, ontological analysis indicates that there are 3 kinases (Pkm2, Mapk8, and Adk, p value = 0.07) and 9 genes involved in metabolism (includes the 3 kinases mentioned above plus Arbp, Usp29, Atp6v1d, Ptpre, and Picalm, p value = 0.04), suggesting key Myc targets in precursors are involved in signaling and cell metabolism. Of the shared genes upregulated (putative Myc suppressed genes), ontological analysis found cellular receptors (p value = 0.01), mostly associated with differentiation including Wnt5a. Wnt signaling more generally was also upregulated specifically in the cerebellum, suggesting Myc may not only be a downstream effector of Wnt, but also regulate Wnt expression.

## DISCUSSION

Although we and others have found very widespread chromatin and transcriptional functions for Myc proteins [15, 16, 19, 52-55], in our expression array studies here on control and *myc* DKO brain tissues, we found relatively modest levels of changes in gene expression and in some domains relatively few genes had altered expression with loss of *myc*. This is somewhat surprising given the striking severity of the DKO phenotype, but it may be reflective of the complexity of cell types in the developing brain (even when divided into fore-, mid-, and hindbrain) compared to more homogeneous cell lines used in previous studies. Nonetheless specific patterns of changes clearly emerged from ontological analyses. We observed that a key function for *myc* in NSC in the developing brain is to maintain overall cellular metabolism with particularly important roles in ribosome biogenesis and nucleotide metabolism.

We also observed an ontological cluster of downregulated mitotic genes that was highly significant, supporting a potential novel role for *myc* in regulating mitosis of normal NSC. Remarkably no mitotic genes were upregulated by loss of Myc. Recently Myc-induced genomic instability has been shown to be due to at least in part to mitotic dysfunction [56], consistent with our findings, and together suggesting normal Myc levels, not too high or too low, are essential for mitosis. Since we have also previously observed abnormal, widespread chromatin changes with loss of *myc* in NSC [16], this altered chromatin state may in addition contribute to problems going through mitosis in *myc* DKO NSC due to premature chromatin condensation in G2 or to failure to decondense late during mitosis. We also observed a decrease in mitotic cells throughout the developing DKO brain ranging from 50% to 2-fold depending on the region, but it remains unclear if the few mitotic DKO NSC are arrested and unable to undergo normal mitosis, or if they can achieve a normal mitosis. Some degree of mitotic arrest in the DKO NSC is of interest, especially as *myc* overexpression has been associated with G2 arrest [57] suggesting *myc* levels may be particularly important for G2/M. Although *myc* has previously been linked to mitosis, that study demonstrated that high levels of *myc* disrupted normal mitosis [58]. Since *myc* has been postulated to dissociate from chromatin during mitosis [59], it may be that during late G2 and early M phases, Myc orchestrates a transcriptional program that then normally carries cells through mitosis. Alternatively some level of Myc still associated with chromatin may directly regulate mitosis during mitosis.

In terms of region-specific brain growth, our model argues that c-, N-, and L-*myc* have both unique and combinatorial roles in directing regional growth of the murine brain. For example, L-*myc* is the prime candidate for the *myc* family member directing midbrain growth, a function that may have remained obscured in the constitutive L-*myc* KO mice due to redundancy with or compensation by N-*myc*. Our findings also suggest that *myc* gene levels and expression patterns may contribute to the differences in relative domain (fore, mid, and hindbrain as well as cerebellar) mass in different species and to brain evolution. For example, echolocators, which are unique in that their cerebellum consists of approximately 20%, instead of the more common 10%, of total brain mass, may have higher total *myc* expression levels or longer periods of *myc* expression during cerebellar development [60]. In addition in organisms with a relatively simpler brain structure, one might predict less complexity in *myc* function, and indeed in *Drosophila Melanogaster*, for example, there is only one *myc* gene, *dmyc* [61].

We did not observe any changes in cell survival and no clear apoptosis-associated gene expression changes were evident in the DKO. Thus, at least c- and N-*myc* appear largely dispensable for NSC survival *in vivo*. This sharply contrasts with HSC, where a dominant phenotype of the c- and N-*myc* DKO is apoptosis manifesting with dramatic changes in apoptosis-related gene expression [25]. Together these findings suggest the intriguing possibility that c and N-*myc* may have fundamentally distinct roles in NSC and HSC. Recent murine “placental rescue” [62] experiments point to the key role of c-*myc* in development being hematopoiesis, suggesting N-*myc* plays the primary role in development of the embryo itself. Consistent with this, mutation of N-*myc* in humans causes FS with its very large range of birth defects in a host of tissues[63]. Interestingly the range of defects in constitutive N-*myc* KO mice are remarkably similar to that of FS [64].

It is notable that we observe far more genes upregulated in the DKO brain than downregulated. We have also observed this pattern in gene expression studies in cultured NSC and in ESC (data not shown) [65]. While *myc* has been shown to have a repressive function through Miz1 [66], it has always been widely assumed that *myc* nonetheless is predominantly a transcriptional activator. However, growing evidence suggests that repression by *myc*, direct and/or indirect, is likely to be equally or more important than activation. Consistent with this idea is the notion that a key role for *myc* in iPS cell formation is suppression of fibroblast specific gene expression [34]. In iPS cells *myc* appears to potently enhance reprogramming, suggesting an important role in cell fate. In addition to Miz1, another potential mechanism of repression by *myc* include recruitment of DNMT3A [67].

Enhancing our understanding of *myc* transcriptional function during normal development and in normal stem cells provides a window into how excess *myc* may cause tumorigenesis, potentially providing additional targets to treat *myc*-related tumors. New inhibitors targeting Myc interaction with Max show promise [68], but it may also prove fruitful to target functions downstream of Myc such as transcriptional or chromatin events as well as cellular states maintained by Myc including cellular “stemness”. Consistent with this idea, Myc has recently been linked with maintaining an aberrant pluripotent state in tumor stem cells including those of glioma [69] and medulloblastoma [70, 71]. Forcing changes in chromatin that in turn lead to differentiation of cancer cells that have excess Myc may be a new method of treatment that does not rely upon targeting Myc itself. Future studies on the differences in Myc-regulated chromatin and gene expression between tumor cells and their cognate normal cells of origin should shed additional light on tumorigenesis and potential new chromatin-based treatments.

## MATERIALS AND METHODS

### Animals

The studies involved knockout mice were approved by UC Davis IACUC.

Breeding pairs of nestin Cre- N-*myc*^(FL/FL)^ [22]; c-*myc*^(FL/FL)^ [49] and nestin Cre+ N-*myc*^(FL/WT)^ c-*myc*^(FL/FL)^ were set up to generate the four genotypes Nestin Cre- N-*myc*^(FL/FL)^ c-*myc*^(FL/FL)^, Nestin Cre- N-*myc*^(FL/WT)^ c-*myc*^(FL/FL)^, Nestin Cre+ N-*myc*^(FL/FL)^ c-*myc*^(FL/FL)^, and nestin Cre+ N-*myc*^(FL/WT)^ c-*myc*^(FL/FL)^. Genotypes of animals were determined via PCR using primer sets for Cre, N-*myc* and c-*myc*. Primer sequences were as follow: Cre1: GCC TGC ATT ACC GGT CGA TGC AAC GA; Cre2: GTG GCA GAT GGC GCG GCA ACA ACC ATT; N-*myc*1: GTC GCG CTA GTA AGA GCT GAG ATC; N-*myc*2: GGC ACA CAC CTA TAA TCC CAG CTA; N-*myc*3: CAC AGC TCT GGA AGG TGG GAG AAA GTT GAG CGT CTC C; c-*myc*1: GCC CCT GAA TTG CTA GGA AGA CTG; c-myc2: CCG ACC GGG TCC GAG TCC CTA TT. Cre1 and 2 primer sets amplified a 700bp band from Cre insertion. The flox specific band for N-*myc*1, 2 and 3 primers is 260bp, the wild-type band is 217bp, and the deletion band is 350bp. The flox specific band for c-*myc*1 and 2 primer set is 500bp, and the wild-type band is 400bp. A separate primer set to detect c-*myc* deletion, c-*myc*DS: TCG CGC CCC TGA ATT GCT AGG AA, and c-*myc*DA: TGC CCA GAT AGG GAG CTG TGA TAC TT, were used and amplifies a band at ~750bp.

### Immunohisto- and cytochemistry

All E12.5 embryos were immersion-fixed overnight in fresh, buffered 4% paraformaldehyde, paraffin embedded and cut (12μm) sagittally. Pregnant female were anesthetized with 150mg/kg ketamine and 16mg/kg xylazine, then E17.5 embryos were removed individually and perfused with buffered 4% paraformaldehyde. Brains were removed and fixed overnight in fresh, buffered 4% paraformaldehyde, then cryopreserved and cut (12μm) sagittally. For immunostaining, E12.5 embryo sections were deparaffinized then rehydrated and 30mM sodium citrate treated for 10 minutes at 95°C, while E17.5 frozen brain sections were post fixed in −20°C Acetone for 10 minutes followed by three 5 minutes PBS wash. All sections were blocked in 10% normal goat serum in PBS for 1 hour at room temperature then incubated in primary antibody overnight at 4°C. Antibodies used include anti-BrdU (Chemicon MAB3424, 1:150), anti-PhosphoH3 (Upstate 06-570, 1:200), and anti-TubulinßIII (TUJ; Covance PRB-435P, 1:150). The next day sections were washed in PBS three times 10 minutes each, then incubated in secondary antibody for 2 hours at room temperature. Alexa Fluor Goat α Rabbit IgG λ488 (Invitrogen A11008) and Alexa Fluor Goat α Mouse IgG λ546 (Invitrogen A 11003) were used at 1:1000 dilution. Sections were washed again in PBS three times 10 minutes each, then mounted in Vectashield mounting medium with DAPI (VECTOR H-1200). Anti-BrdU staining was conducted on embryos from pregnant females who were IP injected with BrdU (Sigma B5002, 150μg/g) following 2-4 hours BrdU incorporation periods. TUNEL staining was performed using the DeadEnd Fluorometric TUNEL System Kit (Promega G3250) as directed.

### Microarray and Quantitative Real-Time PCR

Cortex, midbrain and cerebellum were dissected from brains of freshly sacrificed E17.5 embryos. Total RNA was isolated from these brain areas using RNeasy Mini Kit (Qiagen 74134) with DNaseI digestion (Invitrogen 18068-015) performed post RNA extraction. Quality of RNA was checked and cRNA was produced by UC Davis Gene Expression Analysis facility for hybridization to Sentrix Mouse Ref-8 Expression microarray. qPCR was performed on total RNA from the same batch sent off for array. Samples were processed using Express SYBR Green qPCR Supermix (Invitrogen A10314) according to manufacture protocol. All *q*PCR data were generated using ß-Actin as reference (house-keeping) gene. This gene was chosen basted on microarray results, which showed no significant expression variation between control and double knockout brains. Primer efficiency was tested by generating standard curves for all primers against each of the 3 brain areas. Cycling was conducted as follows: pre-incubation at 95°C for 5 minutes, 45 cycles of amplification at 95°C for 10 seconds, 60°C for 20 seconds, and 72°C for 30 seconds, followed by one cycle of melting curve at 95°C for 5 seconds, 65°C for 1 minute and 97°C continuous.

### Cell Quantification

Ventricular zone of E12.5 and E17.5 embryo cortex were mapped out according to comparable Nissl stained sections and the mouse brain atlas. Ventricular zone size varied depending on size of cortex. Entire ventricular zone was divided into ten sections, lengthwise, but only cells in the middle eight sections were counted. Total number of cells was obtained by counting all the DAPI positive cells. Number of BrdU and Phospho H3 cells was obtained by counting the TRITC and FITC, respectively, positively stained cells. Percentage of BrdU and Phospho H3 cells was calculated by dividing the total number of BrdU or Phospho H3 cells over the total number of cells in the ventricular zone. Furthermore, for Phospho H3 stained sections a separate percentage of positively stained cells were calculated by combing the subventricular and intermediate zone.

## SUPPLEMENTAL FIGURES


