# Supplementary material for: c-myc and N-myc promote active stem cell metabolism and cycling as architects of the developing brain
Source: Oncotarget. 2010 Jun 4;1(2):120–30. doi: 10.18632/oncotarget.116 (PMC2907900; doi:10.18632/oncotarget.116)
Supplement: Supplementary file 1 [file oncotarget-01-120-s001.pdf]

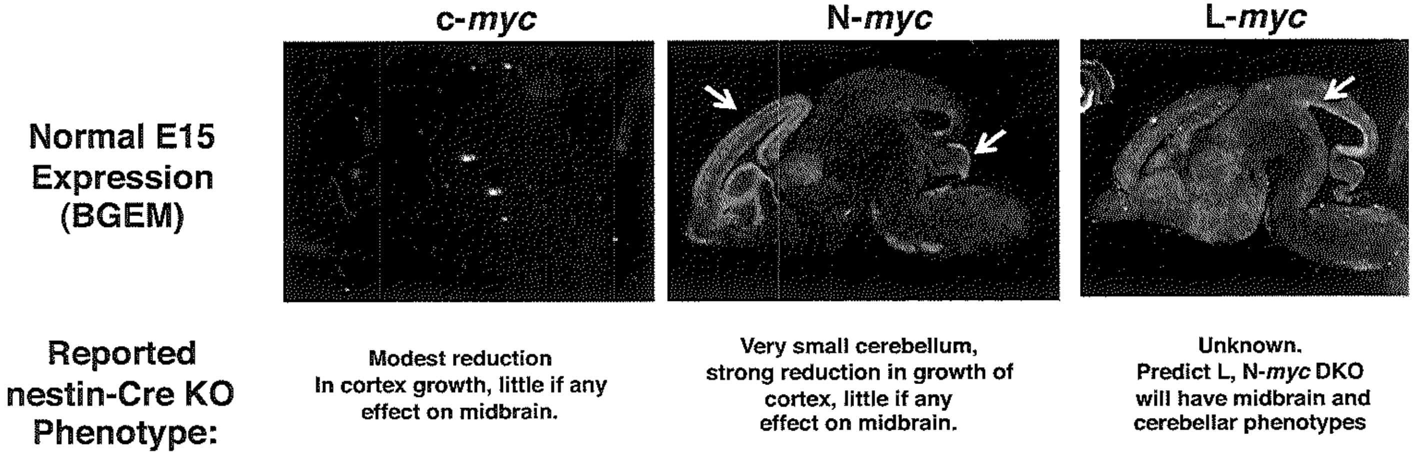

LEGEND:

Myc family gene expression patterns correlated with phenotypes. Top. Normal expression patterns at E15.5 of the 3 main Myc genes (BGEM). White arrows indicate domains of high expression. Bottom. Published nestin-cre mediated KO phenotype, if any, or predictions.

Figure S1

Wey and Knoepfler

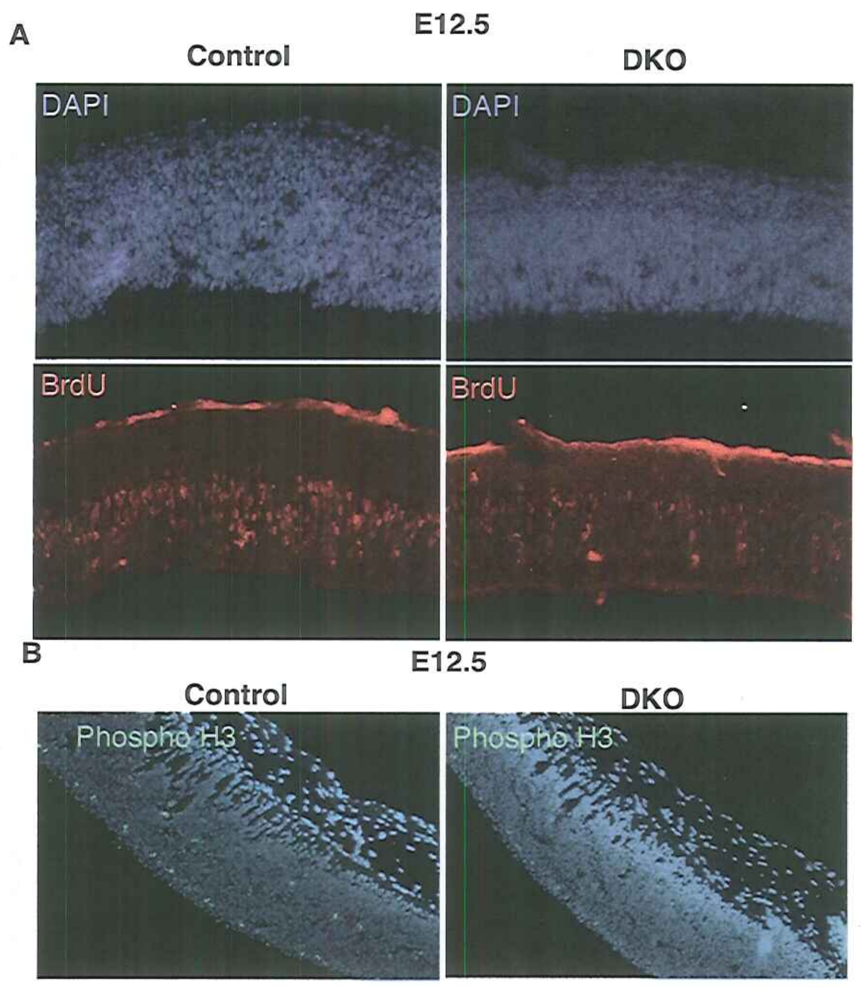

LEGEND:
Loss of Myc decreases NCS cell cycling in the forebrain at E12.5. (A) Control and DKO sagittal sections stained for BrdU (red) and DAPI (blue) following a 2 hr label. (B) Control and DKO sagittal sections stained for DAPI (blue) and the mitotic marker phosphoH3 (green).

Figure S2
Wey and Knoepfler

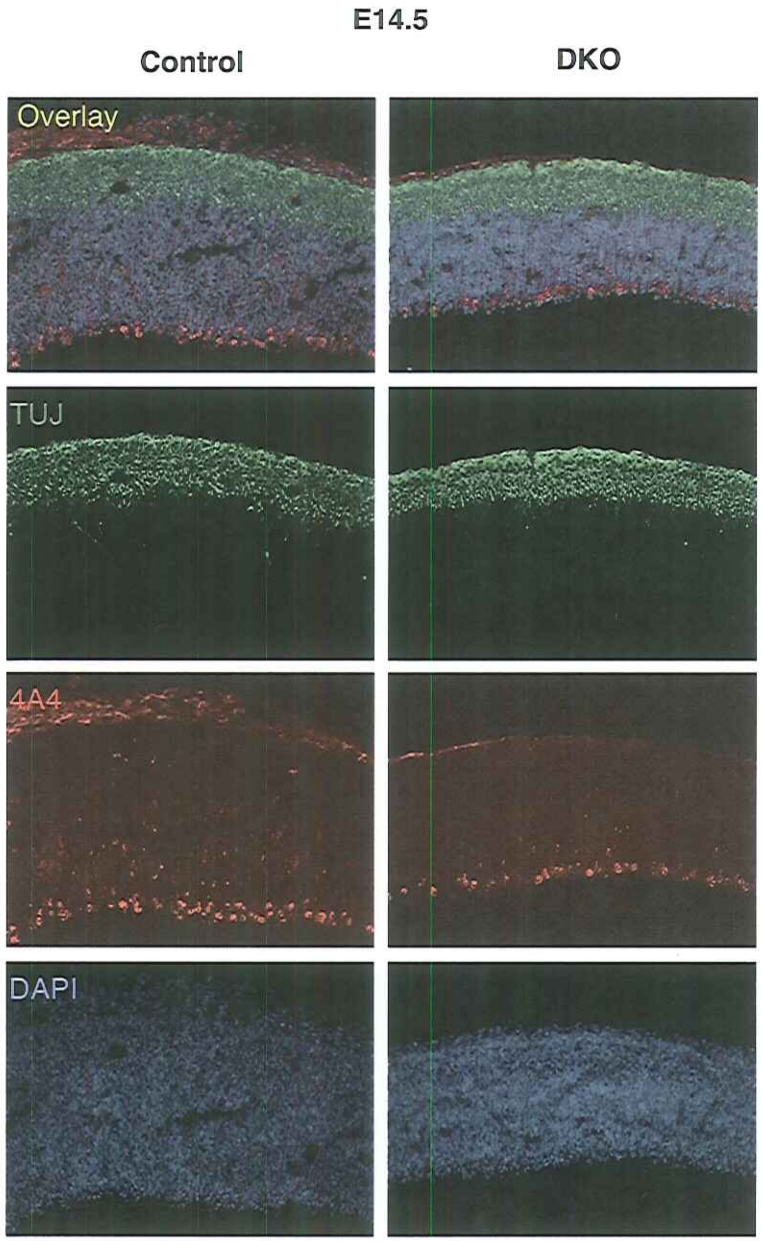

Figure S3
Wey and Knoepfler

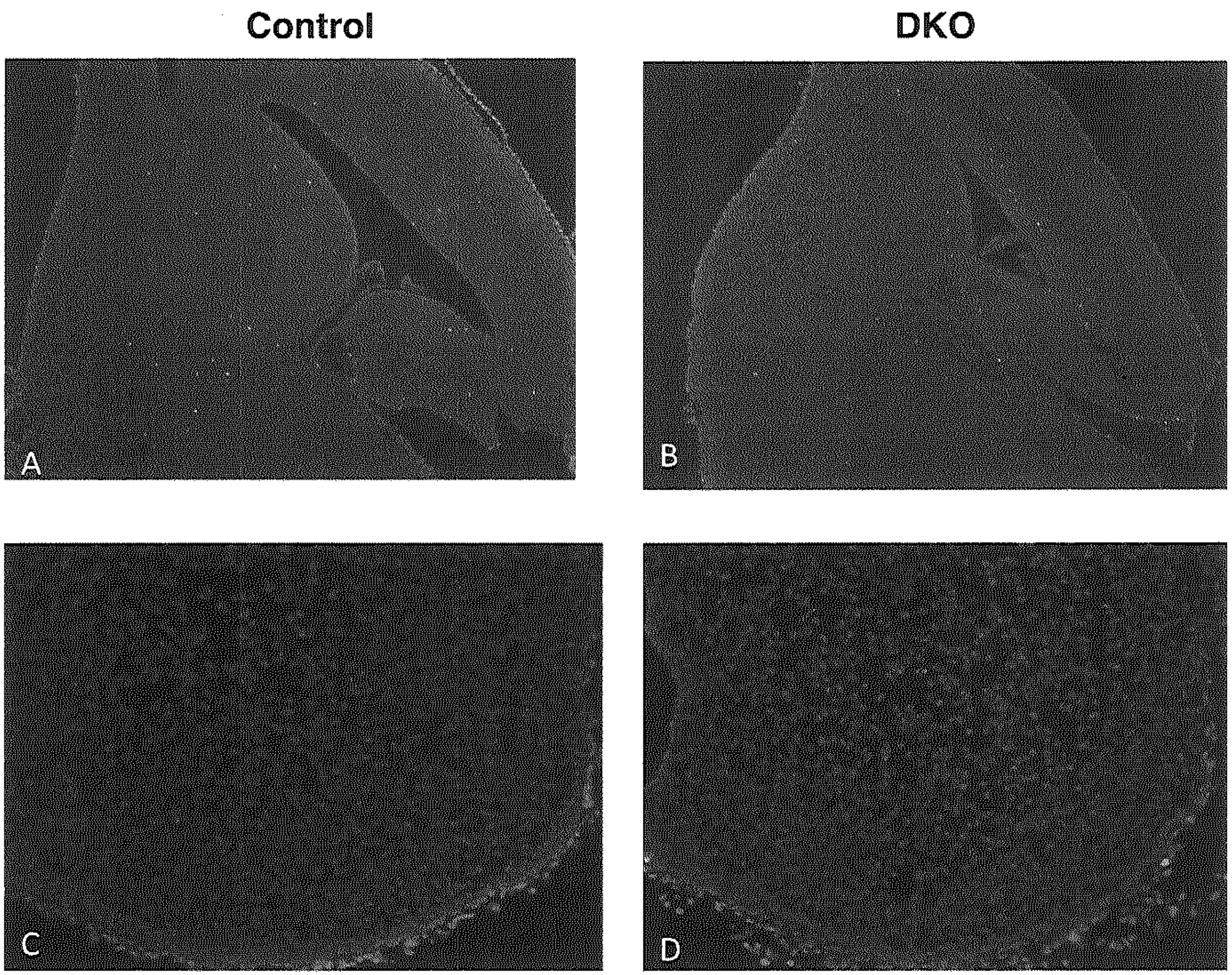

Legend: TUNEL staining of E17.5 brain. (A-B) lateral ventricle, (C-D) cerebellum. DAPI (Blue), TUNEL (green).

Figure S4
Wey and Knoepfler

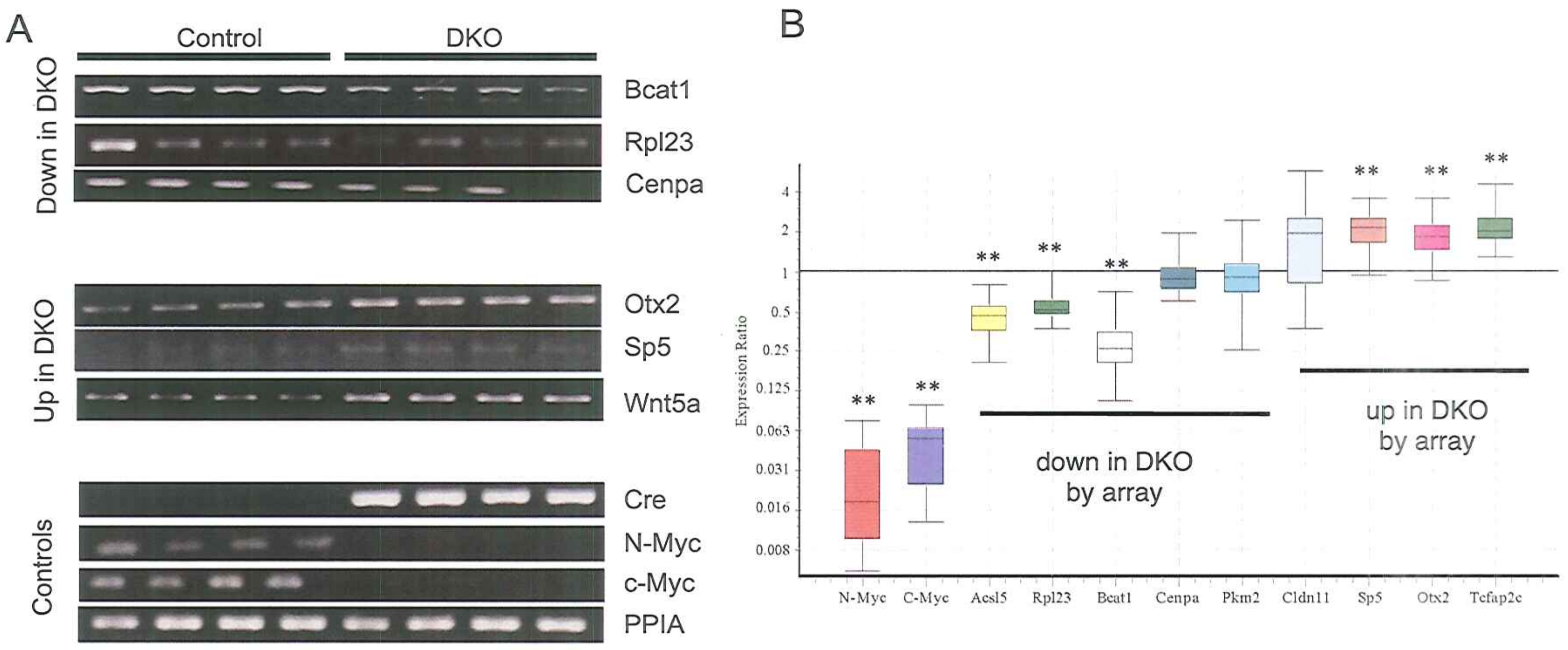

Legend: RT-PCR on c- and N-myc and Cre mRNA levels as well as those of putative genes with altered expression In the DKO forebrain by microarray. Data are the ratios of DKO/Control. (A) and (B) are conventional and q RT-PCR respectively. Error bars are standard deviations.** p value < 0.005.

Figure S5
Wey and Knoepfler

Down in all 3 domains

	Forebrain	Midbrain	Hindbrain
1500012F01Rik	1.953545	1.330658	1.799071
2410015N17Rik	1.380956	1.320027	1.608744
2610206B13Rik	1.68608	1.687102	1.681078
2610528E23Rik	1.389473	1.326666	1.320036
2810417H13Rik	1.832888	1.369072	2.944132
Adk	1.652339	1.4379	1.51814
AI838661	1.34915	1.571047	1.650312
Akr1c19	2.58958	2.173496	1.547631
Arbp	1.45569	1.39505	1.414541
Atp6v1d	1.487886	1.354029	1.306473
C77032	1.453379	1.30675	1.431214
Fbxl3a	1.75876	1.573825	1.556751
Ldh1	1.374228	1.361858	1.505528
LOC544988	2.659666	2.932711	1.993356
LOC545007	2.632969	2.267968	2.147624
LOC545013	2.181864	2.174951	1.502957
Mapk8	1.389015	1.352758	1.304919
Picalm	1.914126	1.923121	1.581222
Pkm2	1.998208	1.608084	1.756061
Ptpre	1.517021	1.394027	1.513023
Rcl1	1.411821	1.358058	1.420385
S100a8	1.359795	1.41392	2.362404
Usp29	1.540584	2.214131	1.464458

Up in all 3 domains

	Forebrain	Midbrain	Hindbrain
1700009P17Rik	0.70	0.76	0.69
4933427D14Rik	0.60	0.47	0.73
Atbf1	0.75	0.69	0.69
BC065120	0.61	0.53	0.65
Cap1	0.61	0.57	0.59
Col1a2	0.73	0.68	0.54
Cops8	0.50	0.49	0.48
D14Ertd449e	0.57	0.59	0.49
Esd	0.72	0.62	0.70
H2afj	0.72	0.65	0.72
LOC73072	0.56	0.43	0.60
Mrc1	0.77	0.71	0.52
Ptprt	0.73	0.76	0.76
Rgs5	0.56	0.60	0.57
Wnt5a	0.67	0.76	0.55
Zcchc3	0.70	0.71	0.73
Zfp330	0.67	0.62	0.71

Legend: Mean array data (Control/DKO ratio) for genes with expression changes in fore, hind, and midbrain.

Figure S6
Wey and Knoepfler
